# Prognostic value of reduced E-cadherin expression in breast cancer: a meta-analysis

**DOI:** 10.18632/oncotarget.14860

**Published:** 2017-01-27

**Authors:** Zhan Li, Songcheng Yin, Lei Zhang, Weiguang Liu, Bo Chen

**Affiliations:** ^1^ Department of Breast Surgery, The First Hospital of China Medical University, Shenyang, Liaoning 110001, China; ^2^ Department of Surgical Oncology, The First Hospital of China Medical University, Shenyang, Liaoning 110001, China

**Keywords:** E-cadherin, breast cancer, prognosis, biomarker, meta-analysis

## Abstract

The prognostic value of E-cadherin expression in patients with breast cancer has been studied for years, yet results remain controversial. We thus performed a comprehensive evaluation of the association between E-cadherin expression and prognosis through a meta-analysis. The databases PubMed, Embase and Cochrane Library were searched. A total of 7,353 patients from 33 studies were subject to final analysis. The results showed there was a significant association between reduced expression of E-cadherin and overall survival (OS) (HR 1.79, 95% CI 1.41–2.27) and disease-free survival (DFS) (HR 1.62, 95% CI 1.31–1.99) in breast cancer. Downregulated expression of E-cadherin significantly correlated with tumor histological grade (OR 1.44, 95% CI 1.06–1.96), TNM stage (OR 2.44, 95% CI 1.75–3.41), tumor size (OR 1.38, 95% CI 1.18–1.60), lymph node status (OR 1.55, 95% CI 1.15–2.10), and progesterone receptor status (OR 1.44, 95% CI 1.10–1.88).This meta-analysis suggested that reduced E-cadherin expression might be a predictor of a poorer prognosis and could be a potentially new gene therapy target for breast cancer patients.

## INTRODUCTION

Among women in the world, breast cancer is the most common cancer with an estimated 1.67 million new cases diagnosed (25% of all cancers) and it was the most frequent cause of cancer death (522,000 deaths, 14.7% of total) in 2012 [1]. Although comprehensive treatment is available,, including radical surgery and adjuvant therapy, the prognosis of breast cancer patients is still far from optimistic [2]. Several common clinicopathological parameters, which include tumor size, lymph node status, histological grade, estrogen receptor (ER), progesterone receptor (PR) and human epidermal growth factor-2 (HER-2) have been extensively applied in the clinic [3]. However, they do not accurately predict an individual's prognosis [4]. It is imperative to explore new prognostic factors to guide treatment and ameliorate survival rates for breast cancer patients [4].

E-cadherin is an essential intercellular adhesion molecule that combines with catenins to form an E-cadherin/β-catenin/α-catenin complex, which is further linked to the actin cytoskeleton [5]. It plays an important role not only in mediating stability of cell adhesion and cell polarity but also in maintaining the integrity of structure and function in epithelial tissues [6, 7]. Downregulated expression of E-cadherin destroys the intracellular junction and thus epithelial cells acquire the ability to migrate. Consequently, decreased expression of E-cadherin facilitates tumor invasion and metastasis [8, 9]. Reduced expression of E-cadherin caused by oncocytes involves several molecular mechanisms: *CDH1* gene mutation, *CDH1* promoter hypermethylation, suppression of RNA transcription, and matriptase activation [10].

It has been reported by Rakha *et al*. that downregulated expression of E-cadherin was correlated with poor survival in a study of 1,516 breast cancer patients [11]. However, Gillett *et al*. assessed the aberrant expression of E-cadherin in 470 cases of infiltrating ductal cancer (IDC) and concluded that low-expression of E-cadherin was a favorable prognostic factor [12]. Wang *et al*. found there was no relationship between E-cadherin expression and prognostic [13]. Therefore, we conducted a meta-analysis to evaluate the association between E-cadherin low-expression and overall survival (OS), disease-free survival (DFS), and clinicopathological parameters in breast cancer.

## RESULTS

### Search results

**Figure 1:**
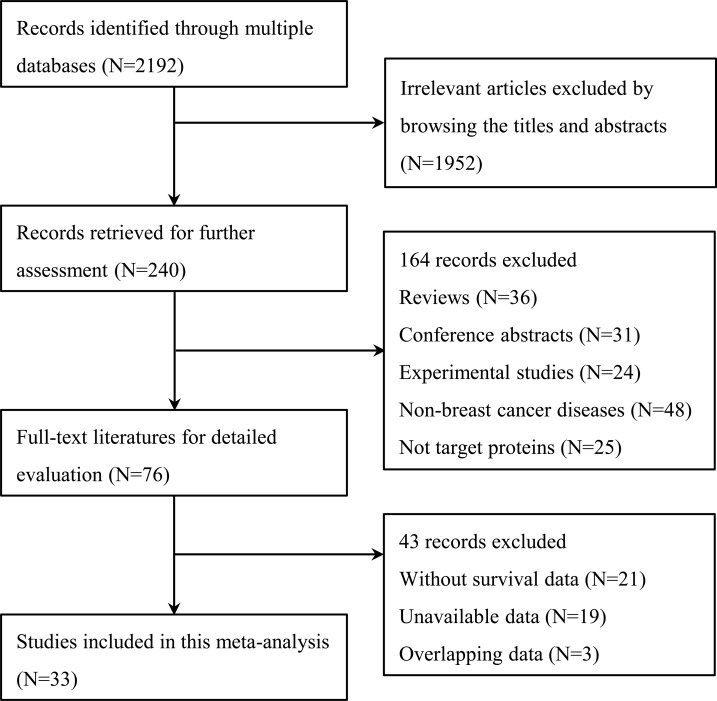
Flow diagram of the study selection process

A total of 2,192 citations were potentially identified for inclusion using the described search strategies. Through reviewing the title and abstracts, 1,952 papers were excluded. Subsequently, an additional 164 records were excluded for the following reasons: They were reviews, conference abstracts, and experimental studies; the source of the tissue was not breast cancer; and, the target protein was not E-cadherin. We then systematically read the full text of the remaining 76 articles and filtered out an additional 43 papers. Among the excluded papers, 21 studies were not associated with survival, 19 studies had no sufficient survival data to analyze and three studies had overlapped data with other published trials. Ultimately, 33 studies [11–43] were included (Figure 1).

### Study characteristics

The fundamental features of the identified articles are shown in Table 1. The total number of patients was 7,353, ranging from 29 to 1,516 in any one study with mean ages of 46–60 years. These studies were published between 1994 and 2016. For the prognostic indicator of reduced E-cadherin expression in breast cancer, 15 articles reported both OS and DFS, 10 articles reported OS, and eight articles reported DFS.

**Table 1:** Characteristics of studies included in our meta-analysis

Author	year	country	Mean age (year)	stage	N	location	Median Follow-up (month)	Cut-off value	Method	Survival	HR estimated	NOS
Asgeirsson	2000	Iceland	58	NR	108	M,	71	50%	IHC	DFS	HR	9
Yu	2015	China	NR	I-III	169	M, C	63.5	165	IHC	DFS, OS	Curves	7
Pedersen	2002	Norway	55	I-IV	61	M	79	5%	IHC	OS	Curves	7
Siitonen	1995	Finland	60	I-IV	109	NR	51	10%	IHC	DFS	A	7
Charpin	1998	France	55	I-III	179	M	67	4%	IHC	OS	Curves	8
Kashiwagi	2010	Japan	58	I-III	574	M	45.7	30%	IHC	DFS, OS	Curves	7
Wang	2015	China	47	I-III	571	M	54	Scores ≤ 99	IHC	DFS, OS	A	8
Shi	2015	China	51	NR	96	M, C	65.2	28%	IHC	OS	HR	9
Pang	2013	China	46	I-III	170	M	75	Scores ≤ 3	IHC	DFS, OS	HR	8
Wang	2014	China	54	I-III	29	NR	50	25%	IHC	DFS, OS	A	8
Liu	2014	china	51	NR	100	C	65.4	28%	IHC	OS	HR	8
Yang	2015	China	NR	NR	125	M	89	Scores < 6	IHC	DFS, OS	HR	7
Bankfalvi	1999	Germany	NR	I-IV	55	M	7	75%	IHC	DFS, OS	Curves	7
Heimann	2000	America	57	NR	168	NR	168	25%	IHC	DFS	HR	9
Pistelli	2014	Italy	54	I-III	81	M	52.4	30%	IHC	DFS, OS	HR	8
Gillett	2001	UK	53	III	470	M, C	NR	Scores ≤ 1	IHC	DFS, OS	A	6
Kim	2010	Korea	49	I-IV	98	M, C	67.8	70%	IHC	OS	HR	7
Lipponen	1994	Finland	57	I-IV	207	M	171.6	50%	IHC	OS	Curves	6
Zhou	2016	China	NR	I-IV	119	M, C	60	10%	IHC	DFS, OS	Curves	7
Li	2014	China	NR	I-III	250	NR	60	Scores < 3	IHC	DFS	HR	7
Park	2007	Norway	54	I-III	196	M	40	Scores ≤ 3	IHC	DFS	Curves	7
Ricciardi	2015	Italy	59	I-IV	45	M	NR	30%	IHC	OS	HR	7
Zhang	2015	China	50	I-III	408	NR	16	NR	IHC	DFS, OS	A	7
Rakha	2005	UK	53	I-III	1516	M	56	Scores ≤ 1	IHC	DFS, OS	HR	8
Saadatmand	2012	Netherland	57	I-IV	502	M	228	53%	IHC	DFS	HR	8
Szasz	2011	Hungary	60	I-III	197	M	111	NR	IHC	DFS	Curves	6
Brzozowska	2012	Poland	58	I-III	89	NR	113.4	70%	IHC	DFS, OS	Curves	7
Yoshida	2001	Japan	54	I-IV	171	NR	59.2	Scores < 1	IHC	DFS, OS	Curves	7
Eljuga	2012	Croatia	NR	I-III	134	M	NR	Scores ≤ 2	IHC	OS	Curves	7
Kavgaci	2010	Turkey	51	I-III	76	M	93.6	10%	IHC	DFS, OS	Curves	8
Lim	2002	Korea	49	I-III	128	M	58.5	70%	IHC	OS	A	8
Kawahara	1997	Japan	52	I-IV	98	NR	27	Scores ≤ 4	IHC	DFS	Curves	7
Liu	2006	China	49	I-III	54	M	36.5	10%	IHC	OS	A	6


NR, not reported; M, membrane; C, cytoplasm; IHC, immunohistochemistry; DFS, disease-free survival; OS, overall survival; HR: hazard ratio; Curves, extrapolated from Kaplan–Meier curves; A, calculated based on the available information; NOS, Newcastle–Ottawa Scale.

### Impact of reduced E-cadherin expression on OS and DFS

**Figure 2:**
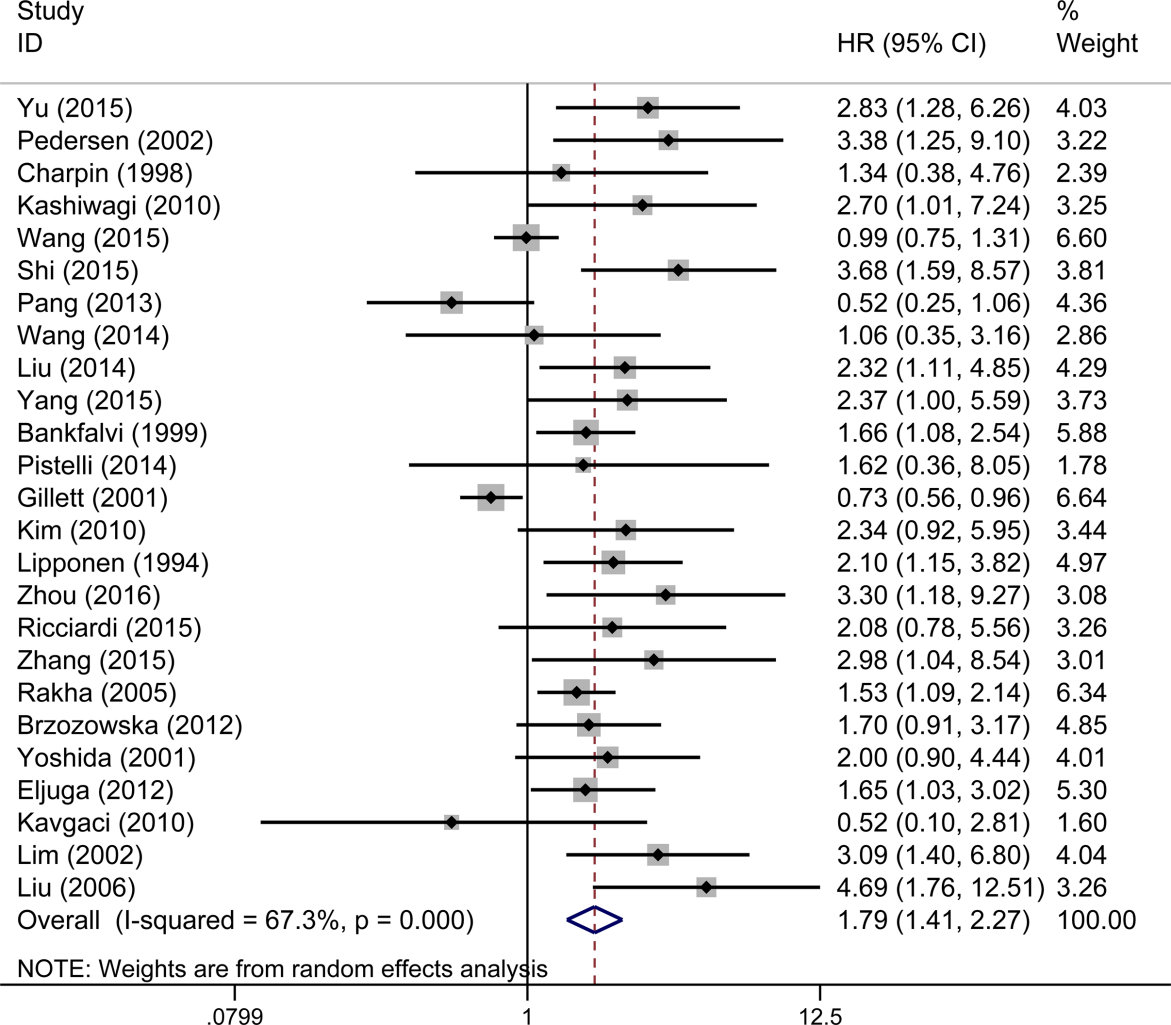
Forest plot of hazard ratio (HR) for the correlation between reduced E-cadherin expression and OS in breast cancer patient

The overall analysis revealed that E-cadherin-negative breast cancer patients had a higher risk of mortality (pooled HR 1.79, 95% CI 1.41–2.27, Figure 2) with heterogeneity (I^2^ = 67.3%, *P* < 0.001). To investigate the source of the OS heterogeneity, subgroup analysis and meta-regression were performed according to publication year, study location, HR estimate, IHC scoring criteria, subcellular localization and pathological types (Table 2). In subgroup analysis, the pooled HRs directly extracted from studies and obtained from Kaplan–Meier curves were 1.77 (95% CI 1.41–2.28) and 1.92 (95% CI 1.55–2.39), demonstrating that reduced expression of E-cadherin was significantly associated with poor OS. Meta-regression analysis indicated that there was no statistically significant difference among subgroups (*P* = 0.637). When the scoring criteria of IHC was taken into consideration, the pooled HR of E-cadherin expression in percentage group was 2.19 (95% CI 1.78–2.70), indicating that there was a significant relationship between reduced expression of E-cadherin and poor OS. In meta-regression analysis, results showed that the difference among subgroups was statistically significant (*P* = 0.024). Pooled HRs were 1.57 (95% CI 1.17–2.10) in the membrane E-cadherin expression group and 2.80 (95% CI 1.92–4.10) in the membrane and cytoplasm E-cadherin co-expression group. Meta-regression analysis showed that there was no statistically significant difference between subgroups (*P* = 0.061).

**Table 2:** Stratified analysis of pooled hazard ratios of breast cancer patients with reduced E-cadherin expression on OS and DFS

Stratified analysis	OS					DFS				
	Pooled HR (95%CI)		Meta-regression *P* value	Heterogeneity		Pooled HR (95%CI)		Meta-regression P value	Heterogeneity	
	fixed	random		I^2^	*P* value	fixed	random		I^2^	*P* value
Year		0.937			0.791	
< 2010	1.32 (1.12,1.56)	1.85 (1.22,2.81)		79.0%	< 0.001	1.38 (1.20,1.59)	1.69 (1.19,2.40)		79.9%	< 0.001
≥ 2010	1.46 (1.23,1.73)	1.77 (1.30,2.41)	57.4%	0.002	1.49 (1.29,1.73)	1.58 (1.21,1.99)	63.2%	0.001
Nation		0.209			0.925	
Asia	1.56 (1.30,1.88)	2.12 (1.45,3.09)		68.9%	< 0.001	1.44 (1.21,1.71)	1.61 (1.13,2.28)		69.6%	< 0.001
Non-Asia	1.27 (1.08,1.48)	1.51 (1.09,2.08)	65.8%	0.001	1.43 (1.27,1.61)	1.63 (1.24,2.14)	74.3%	< 0.001
HR estimate		0.637			0.485	
Directly	1.65 (1.30,2.09)	1.77 (1.41,2.28)		55.5%	0.028	1.67 (1.23,2.26)	1.63 (1.40,1.91)		62.4%	0.009
Calculated	0.99 (0.83,1.19)	1.60 (0.93,2.75)	81.3%	< 0.001	1.13 (0.75,1.71)	0.94 (0.79,1.13)	74.1%	0.004
Curves	1.92 (1.55,2.39)	1.92 (1.55,2.39)	0.0%	0.693	1.93 (1.59,2.34)	1.93 (1.59,2.34)	0.0%	0.512
Scoring criteria		0.024			0.423	
Percentage	2.19 (1.78,2.70)	2.19 (1.78,2.70)		0.0%	0.613	2.11 (1.52,2.92)	2.13 (1.68,2.70)		38.5%	0.135
Intensity	0.93 (0.74,1.16)	1.21 (0.68,2.15)	72.9%	0.011	1.19 (0.76,1.87)	1.09 (0.92,1.30)	79.6%	0.001
Combined	1.22 (1.00,1.48)	1.33 (0.84,2.10)	74.8%	0.003	1.58 (1.20,2.09)	1.47 (1.27,1.70)	63.3%	0.004
Location		0.061			0.031	
M	1.24 (1.09,1.41)	1.57 (1.17,2.10)		71.8 %	< 0.001	1.29 (1.16,1.45)	1.37 (1.07,1.75)		73.8%	< 0.001
C, M	2.80 (1.92,4.10)	2.80 (1.92,4.10)	0%	0.925	3.35 (2.03,5.53)	3.35 (2.03,5.53)	0.0%	0.529
Pathological type						
IDC	1.13 (0,97,1.32)	1.61 (1.09,2.39)		78.1%	< 0.001	1.12 (0.95,1.31)	1.58 (1.03,2.44)		82.4%	< 0.001

**Figure 3:**
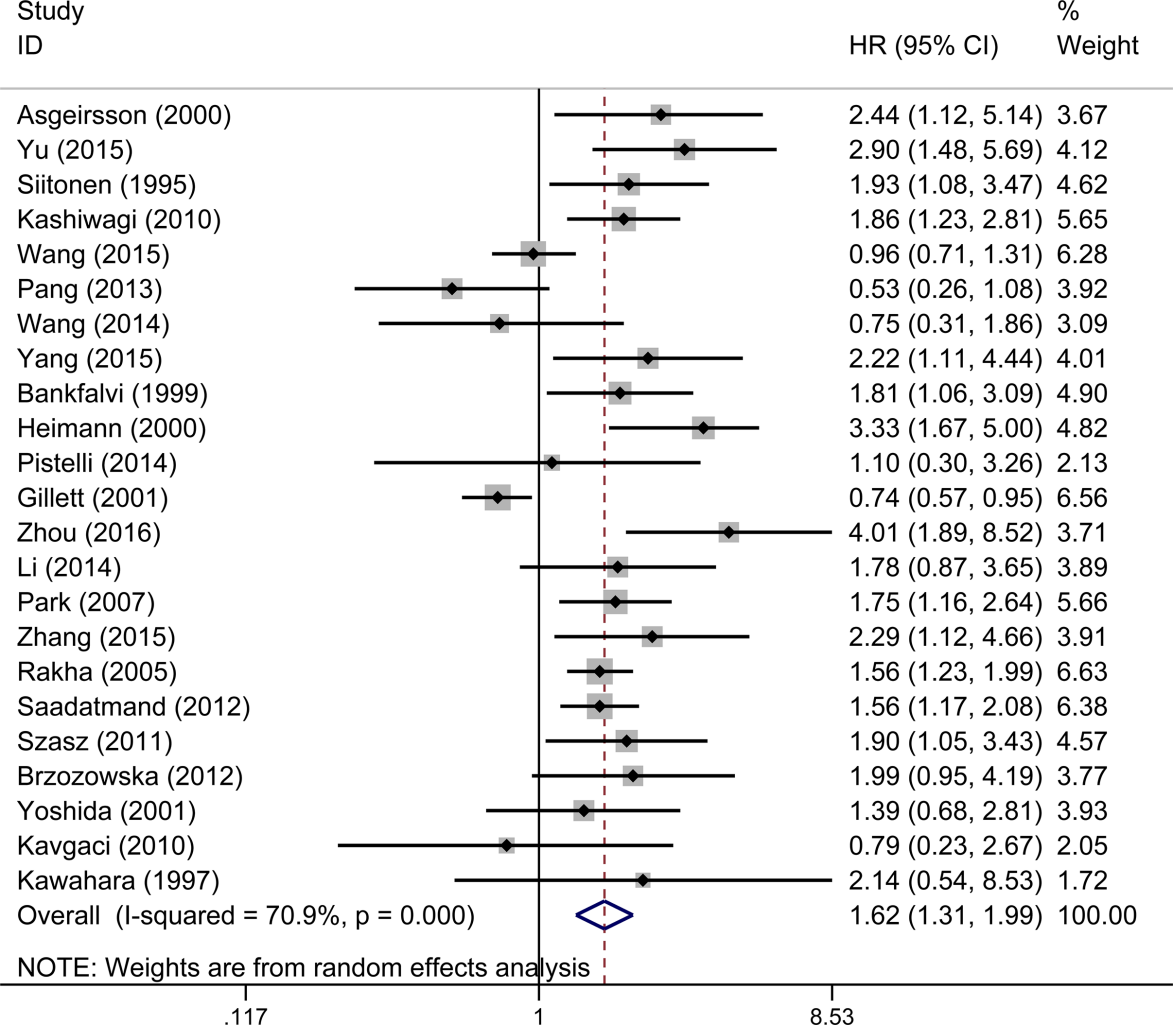
A. Forest plot of hazard ratio (HR) for the association between reduced E-cadherin expression and DFS in breast cancer patient

23 studies evaluated the relationship between decreased E-cadherin expression and DFS, the results showed that E-cadherin low-expression predicted poorer disease-free survival (pooled HR 1.62, 95% CI 1.31–1.99, Figure 3) with significant heterogeneity (I^2^ = 70.9%, *P* < 0.001) of patients with breast cancer. We also conducted subgroup analysis and meta-regression to explain the heterogeneity from six aspects, which are detailed in Table 2. In subgroup analysis, the pooled HRs directly extracted from studies and obtained from Kaplan–Meier curves were 1.63 (95% CI 1.40–1.91) and 1.93 (95% CI 1.59–2.34). Both of them showed that reduced expression of E-cadherin was significantly associated with disease progression. No significant heterogeneity was found in meta-regression analysis (*P* = 0.485). Pooled HRs were 2.11 (95% CI 1.52–2.92) in the percentage group and 1.47 (95% CI 1.27–1.70) in the complex score group. Meta-regression analysis showed that no significant statistical difference was found (*P* = 0.423). The results showed that in the group of membrane location (pooled HR 1.37, 95% CI 1.07–1.75) and group of membrane and cytoplasm location (pooled HR 3.35, 95% CI 2.03–5.53), indicating that downregulated expression of E-cadherin was correlated with poor DFS. Importantly, a significant heterogeneity was observed in meta-regression analysis (*P* = 0.031).

### Evaluation of reduced E-cadherin expression and clinicopathological characteristics

As illustrated in Table 3, E-cadherin low-expression was significantly associated with lymph node (positive vs. negative: OR 1.55, 95% CI 1.15–2.10), tumor size (≥ 2 cm vs. < 2 cm, OR 1.38, 95% CI 1.18–1.60), histological grade (II–III vs. I: OR 1.44, 95% CI 1.06–1.96), TNM stage (T3/T4 vs. T1/T2: OR 2.44, 95% CI 1.75–3.41), and PR status (negative vs. positive: OR 1.44, 95% CI 1.10–1.88). However, no significant correlation was found between E-cadherin low-expression and ER status (negative vs. positive: OR 1.32, 95% CI 0.94–1.84), HER-2 status (≥ 2+ vs. 1+ OR 1.36, 95% CI 0.86–2.16), onset age (≥ 50 vs. < 50 OR 1.03, 95% CI 0.85–1.24), menstrual status (post vs. premenstrual OR 1.20, 95% CI 0.90–1.60), and pathological type (IDC vs. others OR 0.77, 95% CI 0.59–1.00).

**Table 3:** Meta-analysis of reduced E-cadherin expression and clinicopathological features in breast cancer

	No. of studies	Pheterogeneity	I^2^(%)	Effect Model	Pooled OR(95%CI)	*P* Value
Tumor size(≥ 2 vs.< 2)	12	0.734	0.0	Fixed model	1.38 (1.18,1.60)	< 0.001
Age(≥ 50 vs.< 50)	12	0.205	25.0	Fixed model	1.03 (0.85,1.24)	0.706
Histological grade(II/IIIvs.I)	14	0.001	63.6	Random model	1.44 (1.06,1.96)	0.02
TNM stage(T3/T4vs.T1/T2)	7	0.086	45.9	Fixed model	2.44 (1.75,3.41)	< 0.001
Pathological type(IDC vs.Others)	7	0.629	0.0	Fixed model	0.77 (0.59,1.00)	0.054
Menopause status(Post vs.Pre)	6	0.341	11.6	Fixed model	1.20 (0.90,1.60)	0.219
Lymph node status(+ vs.−)	15	< 0.001	72.1	Random model	1.55 (1.15,2.10)	0.005
ER status(− vs.+)	12	0.002	61.8	Random model	1.32 (0.94,1.84)	0.108
PR status(− vs.+)	8	0.399	4.0	Fixed model	1.44 (1.10,1.88)	0.007
Her2 status(≥ 2+ vs.1+)	5	0.197	33.6	Fixed model	1.36 (0.86,2.16)	0.185

### Sensitivity analysis

**Figure 4:**
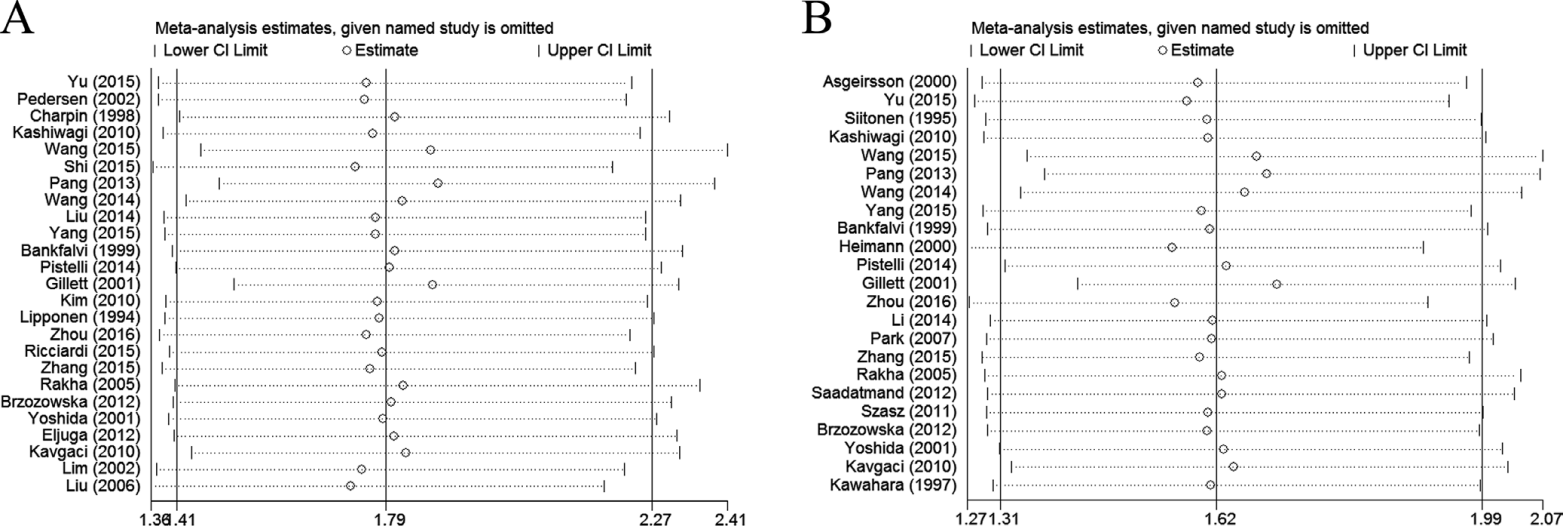
Sensitivity analysis in this meta-analysis (**A**) Sensitivity analysis for the reduced E-cadherin expression with OS. (**B**) Sensitivity analysis for the reduced E-cadherin expression with DFS.

We further performed sensitivity analysis to gauge the stability of our results with respect to OS, DFS, and clinicopathological characteristics. The plots illustrated the robustness of our results because excluding any single study did not significantly influence pooled HRs or ORs (Figure 4).

### Publication bias

**Figure 5:**
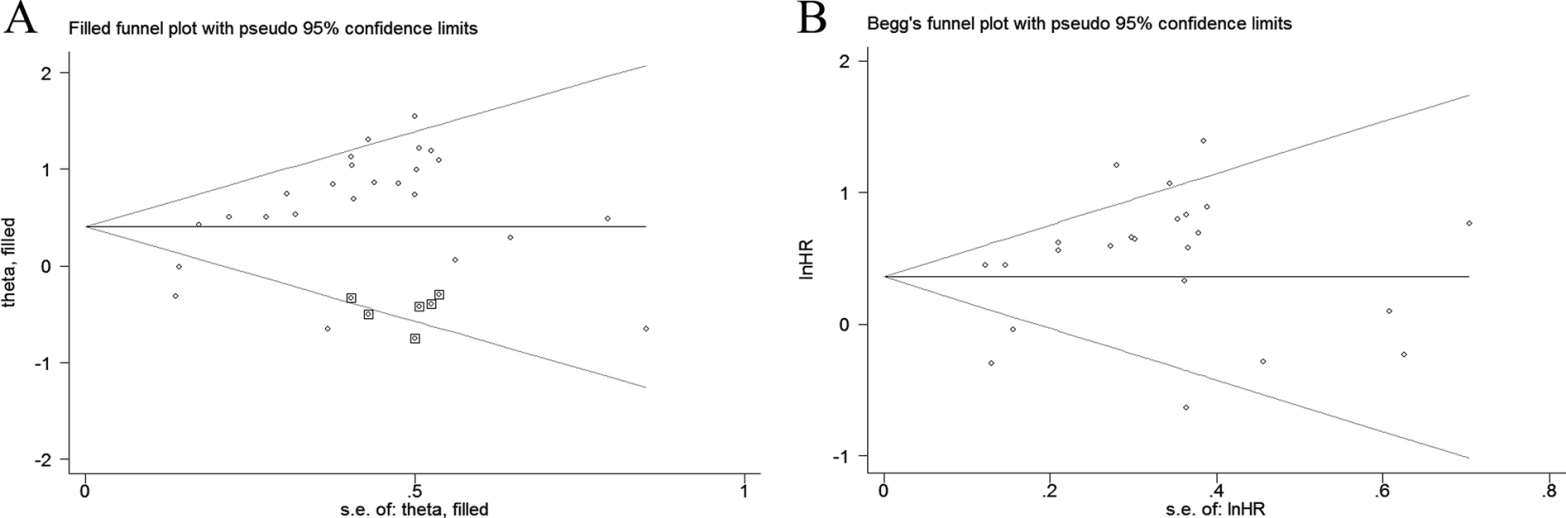
Funnel plot for the assessment of publication bias in this study (**A**) Funnel plot of trim-and-fill analysis for the reduced E-cadherin expression with OS (**B**) Funnel plot for the reduced E-cadherin expression with DFS.

To assess the publication bias in this meta-analysis, we used both Egger's test and Begg's funnel plots. Both of these tests present the potential proof of the asymmetry of investigating the reduced expression of E-cadherin on OS (P_Egger_= 0.001, P_Begg_= 0.388). Trim-and-fill analysis showed that after incorporating six additional articles, the funnel plots were symmetrical and E-cadherin low-expression was positively correlated with poor OS (corrected HR 1.50, 95% CI 1.20–1.87). Meanwhile, the impact of absent E-cadherin expression on clinicopathological characteristics, Egger's test indicated publication bias existed in lymph node metastasis (P_Egger_= 0.048). Trim-and-fill analysis was conducted and the results showed that abnormal expression of E-cadherin was significantly correlated with lymph node metastasis after incorporating two additional articles (corrected OR 1.39, 95% CI 1.04–1.87). Referring to other indicators, no publication bias was found in these articles. Funnel plots are shown in Figure 5.

## DISCUSSION

E-cadherin, a member of cadherin superfamily of transmembrane glycoproteins, is a linker protein of cell-cell junctions [44]. It is well-known that the functional loss of E-cadherin has been viewed as the most important hallmark of epithelial–mesenchymal transition (EMT), which induces tumor cell dissemination and subsequently increases cell migration and invasion [45, 46]. Besides, the absent of E-cadherin expression has an inseparable relationship with resistance of tumor cells to chemotherapy and radiotherapy [47] and causes cancer cells to present apparent properties of cancer stem cells (CSCs) [48]. Many studies have evaluated the association between decreased E-cadherin expression and the prognosis of breast cancer patients. However, the results are inconsistent. We summarized outcomes from a total of 33 individual studies that included 7,353 breast cancer patients. From this analysis we reached the conclusion that reduced E-cadherin expression significantly predicted poor OS and DFS. Furthermore, the downregulated expression of E-cadherin was correlated with tumor size, lymph node status, TNM stage, and histological grade.

E-cadherin is often split into fragments in the cytoplasm, which in theory its functions would not play an inhibitory effect on EMT [46, 49]. As a result, we further performed a subgroup according to the location of E-cadherin expression. The results showed that E-cadherin expression on membrane was significantly associated with OS (HR 1.57, 95% CI 1.17–2.10) and DFS (HR 1.37, 95% CI 1.07–1.75), which demonstrated that the prognostic role of E-cadherin expressed on the membrane is more precise and meaningful. Considering the heterogeneity of intra-tumor, we conduct a subgroup analysis according to the pathological types of breast cancer. In the subgroup of IDC, the pooled HRs showed that reduced expression of E-cadherin was significantly associated with poor OS (HR 1.62, 95% CI 1.08-2.43) and DFS (HR 1.60, 95% CI 1.09–2.34). It's necessary to analyze the heterogeneity of inter-tumor cells as the prognostic values of E-cadherin may quite different in breast cancer stem cell (CSC) subpopulations [48]. However, it's a pity that there is no articles explored the correlation between E-cadherin expression in CSCs and prognosis, and more original research need to be conducted at this field in the future.

When pooling survival data on OS and DFS, we observed significant heterogeneity among articles. Consequently, meta-regression analysis was conducted and it suggested that IHC scoring criteria and subcellular localization might be vital variables associated with this heterogeneity. In subgroup analysis of IHC scoring criteria, the heterogeneity of E-cadherin expression calculated by percentage group was less than 50%, while the other groups had significant heterogeneity. The potential reason could be that compared with the detection method of staining intensity, assessing the percentage of positive cells was more objective and had more practical clinical implications. In subgroup analysis according to locations of E-cadherin expression, heterogeneity was significant in membrane E-cadherin expression group. It may mainly come from the differences in sample sizes, molecular subtypes, demographic or clinicopathologic data of observational studies. Compared with analyses performed in 2006 [50], the advantages of this meta-analysis were not only that it included more studies and subjects to confirm clinical validity but also that it provided more rigorous evidence to support the results. More importantly, we demonstrated that the prognostic role of E-cadherin expressed on the membrane is more meaningful. Furthermore, we assessed the association between E-cadherin expression and clinicopathological characteristics for breast carcinoma patients.

There were limitations in our meta-analysis. First, different primary antibody sources and antibody dilution ratios can lead to differences in IHC sensitivity. Second, there was no uniform scoring criteria to define E-cadherin positive expression. Furthermore, cut-off values defining reduced E-cadherin expression varied from 5 to 70% without an optimal threshold. Third, HRs estimated from available data and obtained from Kaplan–Meier curves were less reliable owing to inaccuracies in the calculation of censored data. Fourth, we had to exclude studies that had no statistical significance because it was difficult to obtain specific data with which to calculate HRs. Finally, meaningful results trended to be published in English, whereas negative ones were more likely to be published in native languages.

In conclusion, our meta-analysis suggest that reduced E-cadherin expression was not only significantly associated with poorer OS and DFS but also correlated with clinicopathological characteristics including tumor size, lymph node status, TNM stage, and histological grade of breast cancer patients. E-cadherin low-expression might be a useful biomarker for predicting poorer prognosis, especially in the location of membrane, and could be a valuable therapeutic target for breast cancer patients.

## MATERIALS AND METHODS

### Search strategy

We searched for studies on E-cadherin expression and its association with breast cancer prognosis in electronic databases: PubMed, EMBASE, and Cochrane library updated to May 15, 2016. Articles were qualified using the following combined keywords: “breast”, “mammary”, “tumor”, “cancer”, “neoplasm”, “E-cadherin”, “E-CAD”, “cadherin-1”, “prognostic”, and “survival”. References from eligible literature were scanned to minimize any deviation caused during the research process.

### Inclusion criteria

Articles were required to meet the following inclusion criteria: (1) patients diagnosed with breast cancer using pathological and histological examinations; (2) E-cadherin expression was detected in primary tumor tissues; (3) full text, original research articles published in English; (4) statistical results that included hazard ratios (HRs) and 95% confidence intervals (CIs) reported directly or calculated from demographic data or survival curves; and (5) independent E-cadherin expression level data. Only studies with more details and larger sample sizes were selected if duplicate data from other articles occurred. Reviews, letters, conference abstracts, and comments were excluded.

### Quality assessment

The Newcastle-Ottawa quality assessment scale (NOS) was applied to estimate the quality of nonrandomized studies, specifically cohort studies by two investigators independently. According to the NOS, three perspectives were assessed: selection, comparability, and outcomes. Scores higher than six were considered high quality.

### Data extraction

Two researchers extracted the following data independently from qualified studies: (1) publication data including authors, year and country; (2) experimental data including tissue origin, location of E-cadherin expression, percentage of E-cadherin positive cells and cut-off values; (3) demographic data including number of subjects analyzed, ages, and follow-ups; (4) clinicopathological data including tumor size, lymph node status, ER, PR, HER-2, menopausal state, pathologic type, histologic type, and TNM stage; and (5) statistical data including survival analysis, HRs and 95% CIs. Inconsistencies were resolved through negotiation and consultation.

### Statistical analysis

In this meta-analysis, HRs with 95% CIs was applied as appropriate values to measure the impact of reduced E-cadherin expression on survival in breast cancer. In some studies, the value of HR and the 95% CI describing OS and/or DFS could be obtained directly. Otherwise, many studies displaying survival rates with *P* values from log-rank tests or Kaplan–Meier survival curves could also be extrapolated using the method of Parmar and Tierney [51]. For the pooled analysis of the correlation between decreased E-cadherin expression and clinicopathological features, odds ratios (ORs) and their 95% CIs were evaluated. Heterogeneity of the studies was evaluated by the Chi-square-based *Q* test and I^2^. I^2^ < 50% and *P* > 0.05 were considered as acceptable heterogeneity, in which case the fixed-effect model test was performed. Otherwise, the random-effect model test was chosen if significant heterogeneity existed (I^2^ > 50% or *P* < 0.05). Subgroup analysis and meta-regression analysis were performed to detect the source of the heterogeneity. Publication bias was assessed using Begg's test and Egger's test. Sensitivity analysis was carried out to evaluate the stability of the pooled results using sequential omission of individual studies. Furthermore, if multivariate and univariate analyses were both obtainable, the former was chosen. All *P* values were two-sided and *P* < 0.05 was considered statistically significant. All statistical analyses were performed with Stata Version 12.0 (Stata Corporation, College Station, TX, USA).
